# Study of the Molecular Mechanisms of the Therapeutic Properties of Extracellular Vesicles

**DOI:** 10.3390/ijms24087093

**Published:** 2023-04-12

**Authors:** Denis N. Silachev

**Affiliations:** A.N. Belozersky Institute of Physico-Chemical Biology, Lomonosov Moscow State University, 119991 Moscow, Russia; silachevdn@genebee.msu.ru

Extracellular vesicles (EVs) are small biological structures that are released by cells and have important roles in intercellular communication. They are enclosed in a phospholipid bilayer and can contain a variety of biomolecules, including proteins, lipids, and nucleic acids. The term “extracellular vesicle” encompasses various subtypes, including exosomes, microvesicles, and apoptotic bodies, which differ in size, biogenesis, and cargo [1]. EVs have become promising therapeutic agents due to their ability to transport biologically active molecules, signaling proteins, and organelles between cells [2,3]. EVs are an ancient mechanism of intercellular communication that have developed among organisms throughout evolution. It is believed that EVs first emerged in prokaryotic cells as a means of sharing genetic material, and later in eukaryotes, where they uphold crucial roles in intercellular communication. It can be assumed that EVs are a universal mechanism for not only inter-cellular, but even inter-kingdom, communication and possibly one of the tools of evolution (see review [4,5]). EVs can mediate horizontal genetic information transfer, which has been observed in a range of organisms, including bacteria, fungi, plants, and animals [6,7,8,9]. EVs also facilitate communication between organisms of different kingdoms, such as bacteria and animals, or plants and animals.

The transfer of EVs between different kingdoms of life illustrates the intricate connections within biological systems and the potential for developing innovative therapeutic strategies that can harness their unique biological properties. EVs from different kingdoms can impact human cells and tissues through their inherent cargo, as well as their potential to serve as vehicles for delivering drugs. Research has demonstrated that plant-derived EVs have the ability to interact with human or animal cells, and significantly affect physiological processes, such as regulating gut function or treating oncological diseases [10,11,12]. Bacteria produce EVs, called outer membrane vesicles (OMVs), which are small spherical structures secreted by many Gram-negative bacteria [13]. OMVs can carry various virulence factors, such as toxins, enzymes, and lipopolysaccharides, which can be delivered to other bacteria or host cells [14]. However, they also have therapeutic potential, as they can be loaded with drugs or vaccines and used for targeted delivery [15]. Fungi-derived EVs have also been demonstrated to have therapeutic potential for modulating the immune system and delivering drugs [16,17].

This Special Issue is devoted to the study of “Molecular Mechanisms of the Therapeutic Properties of Extracellular Vesicles”, and aims to explore the diverse roles and functions of EVs in both normal and pathological conditions. The collection of research and review articles provides an insight into the molecular mechanisms that mediate the therapeutic effects of EVs, including targeted delivery of drugs and vaccines, and the modulation of physiological processes. The articles cover a range of topics, from the role of EVs in bacterial evolution to the specific cargo molecules that mediate their therapeutic effects in animals. We hope that the articles in this Special Issue inspire further research to explore the uncharted potential of EVs in the context of medicine and biotechnology.

Two articles describing the role of bacterial EVs are included in the Special Issue. Based on a unique ongoing study, Laurin D. et al. examined the OMVs released by specific clones of *E. coli* isolated from the long-term evolution experiment, following 50,000 generations of adaptation to glucose minimal medium [18]. It was shown that the bacterial adaptation to the laboratory conditions resulted in the significant rewiring of the OMV production process, with the particle size, LPS and protein content of the OMVs produced by the 50K-evolved clones significantly differing from those of the ancestral strain. Authors concluded that OMV production in *E. coli* is a phenotype that alters during bacterial evolution, and the contribution of OMV-mediated interactions in bacterial adaptation is questioned. Another exciting study has been published by Schaack B. et al., who analyzed microbiota-derived EVs in human blood [19]. Analysis of healthy donors’ red blood cell concentrate-derived OMVs revealed varied amounts of outer membrane protein A (OmpA) and LPS in 85% of the samples, indirectly indicating the presence of microbiota EVs in the human circulating blood, in the absence of barrier disruption in the gut. Subsequent analysis showed the possible interaction of OMVs with monocytes in the circulating blood. This study provides evidence that bacterial OMVs play a crucial role in host–microbiota cellular communication, and analysis of bacterial OMVs should be a part of human EV assessment. The presence of bacterial EVs in human blood is increasingly attracting the attention of researchers, as such EVs are considered potential players in carcinogenesis [20]. Research in this direction can lead to a better understanding of the complex interactions between microbiota and the host, as well as their role in human health and disease.

A considerable amount of current research has been directed toward exploring the healing potential of EVs obtained from MSCs, which has led to a surge of interest in this field of study. The use of MSC-derived EVs expands on previous stem cell therapies, overcoming many of the challenges related to the limited availability, storage, and transplantation costs of live cells. Sanz-Ros et al. reviewed the potential therapeutic role of EVs in various aging-related diseases [21]. EVs have been found to regulate crucial aging-related processes, such as oxidative stress and cell senescence, both in vivo and in vitro. Moreover, EVs, especially those derived from stem cells, have been identified as potential agents for regenerative therapies, with a number of animal models have demonstrated the potency of EVs in the field of neural tissue regeneration. Demyanenko et al. studied approaches to the regeneration of peripheral nerve injury, such as neonatal brachial plexus palsy, which is a significant medical challenge with no effective treatment currently available [22]. The application of MSC-EVs to promote peripheral nerve regeneration was proposed. This approach was tested in rats with sciatic nerve injury, wherein gel-containing MSC-EVs were administered, resulting in reduced muscle atrophy and functional recovery after one month. The positive effects were attributed to the EV-induced neuroprotective mechanisms that decreased apoptotic neuronal death and increased regeneration-associated proteins, as observed in the dorsal root ganglions and damaged nerves. However, a challenging aspect in the study of EVs is the determination of cargo being responsible for their therapeutic effects, including that of neuroprotective nature. In particular, recent research has revealed that MSC-derived EVs protected against cell death caused by calcium ion overload by inducing PI3K/AKT signaling pathways, and the components of this pathway were enriched in the MSC-EV proteome, highlighting its potential as a therapeutic tool [23].

The study by Khandagale et al. focused on the miR profile of EVs from the plasma of pulmonary arterial hypertension (PAH) patients and healthy controls [24]. miR profiling showed 22 down-regulated and six up-regulated miRs in PAH EVs, including miR-26a-5p and miR-486-5p, respectively. The angiogenic and proliferative effects of miRs from PAH EVs were mediated through NF-κB activation, and could be inhibited by silencing miR-486-5p or overexpressing miR-26a-5p. The study suggests that an altered miR profile in PAH EVs could be targeted to reduce pulmonary endothelium activation and restrict angiogenesis. Further studies are warranted to understand the potential of miRs in heterogeneous EVs for PAH treatment.

The ability of EVs to interact with target cells and induce downstream biological effects is determined not only by their cargo, but also by the surface molecules. These molecules play a crucial role in the uptake mechanisms, cargo delivery, and targeting of EVs, and can also serve as biomarkers with potential diagnostic value. Clos-Sansalvador et al. focused on the role of N-glycosylation of MSC-derived EVs in mediating their interaction with endothelial cells [25]. The study found that N-glycosylation in EVs was essential for their interaction with endothelial cells and the subsequent induction of cell migration and angiogenesis. On the contrary, the removal of N-glycans through glycosidase PNGase-F treatment reduced the functional activities of native MSC-EVs, including capture by HUVEC cells and tube-like formation stimulation. Overall, the study emphasizes the importance of N-glycans in MSC-EVs for their interactions with cells and implementation of their functions.

Mustajab et al. provided an informative overview of the potential of EVs as tools for the treatment of COVID-19 [26]. The authors discuss the various ways in which EVs could be used in the development of vaccines and therapeutics, including their potential for delivering antigens and drugs directly to target cells. The article provides a comprehensive review of the current advances regarding EVs in relation to COVID-19. Additionally, the potential obstacles associated with the clinical use of extracellular vesicles and possible solutions have been discussed. This article is a valuable resource for researchers and clinicians interested in the role of EVs in the fight against COVID-19.

Furthermore, non-alcoholic steatohepatitis (NASH) is a rising global health concern and the main reason for liver transplantation. Therefore, there is a pressing need to develop more efficacious therapies to manage the pathology of this condition. In recent years, there has been growing interest in the emerging role of EVs and autophagy machinery in the pathogenesis of NASH. Trifylli et al. reviewed the interplay between autophagy and EVs in the development of NASH, and their potential for novel therapeutic strategies, that could improve clinical outcomes for patients with NASH [27].

In conclusion, research on the molecular mechanisms of the therapeutic properties of extracellular vesicles has provided insights into their potential as promising tools for the treatment of various diseases. Notably, there are impressive prospects for the clinical use of EVs from diverse sources, including stem cells, plants, and bacteria. Progress in the identification and isolation of EVs, as well as the development of clinical application protocols, could guide us to the discovery of novel drugs in the near future. However, extended research and clinical trials are still necessary to fully establish their efficacy, safety, and effectiveness in the treatment of different diseases.

## References

[1] van Niel G., D’Angelo G., Raposo G. (2018). Shedding Light on the Cell Biology of Extracellular Vesicles. Nat. Rev. Mol. Cell Biol..

[2] Murphy D.E., de Jong O.G., Brouwer M., Wood M.J., Lavieu G., Schiffelers R.M., Vader P. (2019). Extracellular Vesicle-Based Therapeutics: Natural versus Engineered Targeting and Trafficking. Exp. Mol. Med..

[3] Zorova L.D., Kovalchuk S.I., Popkov V.A., Chernikov V.P., Zharikova A.A., Khutornenko A.A., Zorov S.D., Plokhikh K.S., Zinovkin R.A., Evtushenko E.A. (2022). Do Extracellular Vesicles Derived from Mesenchymal Stem Cells Contain Functional Mitochondria?. Int. J. Mol. Sci..

[4] Gill S., Catchpole R., Forterre P. (2019). Extracellular Membrane Vesicles in the Three Domains of Life and Beyond. FEMS Microbiol. Rev..

[5] Li D., Yang J., Yang Y., Liu J., Li H., Li R., Cao C., Shi L., Wu W., He K. (2021). A Timely Review of Cross-Kingdom Regulation of Plant-Derived MicroRNAs. Front. Genet..

[6] Ghanam J., Chetty V.K., Barthel L., Reinhardt D., Hoyer P.-F., Thakur B.K. (2022). DNA in Extracellular Vesicles: From Evolution to Its Current Application in Health and Disease. Cell Biosci..

[7] Fang Y., Wang Z., Liu X., Tyler B.M. (2022). Biogenesis and Biological Functions of Extracellular Vesicles in Cellular and Organismal Communication with Microbes. Front. Microbiol..

[8] Urzì O., Gasparro R., Ganji N.R., Alessandro R., Raimondo S. (2022). Plant-RNA in Extracellular Vesicles: The Secret of Cross-Kingdom Communication. Membranes.

[9] Valcz G., Újvári B., Buzás E.I., Krenács T., Spisák S., Kittel Á., Tulassay Z., Igaz P., Takács I., Molnár B. (2022). Small Extracellular Vesicle DNA-Mediated Horizontal Gene Transfer as a Driving Force for Tumor Evolution: Facts and Riddles. Front. Oncol..

[10] Kameli N., Dragojlovic-Kerkache A., Savelkoul P., Stassen F.R. (2021). Plant-Derived Extracellular Vesicles: Current Findings, Challenges, and Future Applications. Membranes.

[11] Tan Z.-L., Li J.-F., Luo H.-M., Liu Y.-Y., Jin Y. (2022). Plant Extracellular Vesicles: A Novel Bioactive Nanoparticle for Tumor Therapy. Front. Pharmacol..

[12] Cai Y., Zhang L., Zhang Y., Lu R. (2022). Plant-Derived Exosomes as a Drug-Delivery Approach for the Treatment of Inflammatory Bowel Disease and Colitis-Associated Cancer. Pharmaceutics.

[13] Jan A.T. (2017). Outer Membrane Vesicles (OMVs) of Gram-Negative Bacteria: A Perspective Update. Front. Microbiol..

[14] Schwechheimer C., Kuehn M.J. (2015). Outer-Membrane Vesicles from Gram-Negative Bacteria: Biogenesis and Functions. Nat. Rev. Microbiol..

[15] Jalalifar S., Morovati Khamsi H., Hosseini-Fard S.R., Karampoor S., Bajelan B., Irajian G., Mirzaei R. (2023). Emerging Role of Microbiota Derived Outer Membrane Vesicles to Preventive, Therapeutic and Diagnostic Proposes. Infect. Agent Cancer.

[16] Freitas M.S., Bonato V.L.D., Pessoni A.M., Rodrigues M.L., Casadevall A., Almeida F. (2019). Fungal Extracellular Vesicles as Potential Targets for Immune Interventions. mSphere.

[17] Rizzo J., Rodrigues M.L., Janbon G. (2020). Extracellular Vesicles in Fungi: Past, Present, and Future Perspectives. Front. Cell. Infect. Microbiol..

[18] Laurin D., Mercier C., Quansah N., Robert J.S., Usson Y., Schneider D., Hindré T., Schaack B. (2022). Extracellular Vesicles from 50,000 Generation Clones of the Escherichia Coli Long-Term Evolution Experiment. Int. J. Mol. Sci..

[19] Schaack B., Hindré T., Quansah N., Hannani D., Mercier C., Laurin D. (2022). Microbiota-Derived Extracellular Vesicles Detected in Human Blood from Healthy Donors. Int. J. Mol. Sci..

[20] Chronopoulos A., Kalluri R. (2020). Emerging Role of Bacterial Extracellular Vesicles in Cancer. Oncogene.

[21] Sanz-Ros J., Mas-Bargues C., Romero-García N., Huete-Acevedo J., Dromant M., Borrás C. (2022). Therapeutic Potential of Extracellular Vesicles in Aging and Age-Related Diseases. Int. J. Mol. Sci..

[22] Demyanenko S.V., Pitinova M.A., Kalyuzhnaya Y.N., Khaitin A.M., Batalshchikova S.A., Dobaeva N.M., Shevtsova Y.A., Goryunov K.V., Plotnikov E.Y., Pashkevich S.G. (2022). Human Multipotent Mesenchymal Stromal Cell–Derived Extracellular Vesicles Enhance Neuroregeneration in a Rat Model of Sciatic Nerve Crush Injury. Int. J. Mol. Sci..

[23] Turovsky E.A., Golovicheva V.V., Varlamova E.G., Danilina T.I., Goryunov K.V., Shevtsova Y.A., Pevzner I.B., Zorova L.D., Babenko V.A., Evtushenko E.A. (2022). Mesenchymal Stromal Cell-Derived Extracellular Vesicles Afford Neuroprotection by Modulating PI3K/AKT Pathway and Calcium Oscillations. Int. J. Biol. Sci..

[24] Khandagale A., Corcoran P., Nikpour M., Isaksson A., Wikström G., Siegbahn A., Christersson C. (2022). MircoRNA in Extracellular Vesicles from Patients with Pulmonary Arterial Hypertension Alters Endothelial Angiogenic Response. Int. J. Mol. Sci..

[25] Clos-Sansalvador M., Garcia S.G., Morón-Font M., Williams C., Reichardt N.-C., Falcón-Pérez J.M., Bayes-Genis A., Roura S., Franquesa M., Monguió-Tortajada M. (2022). N-Glycans in Immortalized Mesenchymal Stromal Cell-Derived Extracellular Vesicles Are Critical for EV-Cell Interaction and Functional Activation of Endothelial Cells. Int. J. Mol. Sci..

[26] Mustajab T., Kwamboka M.S., Choi D.A., Kang D.W., Kim J., Han K.R., Han Y., Lee S., Song D., Chwae Y.-J. (2022). Update on Extracellular Vesicle-Based Vaccines and Therapeutics to Combat COVID-19. Int. J. Mol. Sci..

[27] Trifylli E.-M., Kriebardis A.G., Koustas E., Papadopoulos N., Deutsch M., Aloizos G., Fortis S.P., Papageorgiou E.G., Tsagarakis A., Manolakopoulos S. (2022). The Emerging Role of Extracellular Vesicles and Autophagy Machinery in NASH—Future Horizons in NASH Management. Int. J. Mol. Sci..

